# Gamma Knife Surgery for Residual or Recurrent Craniopharyngioma After Surgical Resection: A Multi-institutional Retrospective Study in Japan

**DOI:** 10.7759/cureus.6973

**Published:** 2020-02-12

**Authors:** Takahiko Tsugawa, Tatsuya Kobayashi, Toshinori Hasegawa, Yoshiyasu Iwai, Shigeo Matsunaga, Masaaki Yamamoto, Motohiro Hayashi, Hiroyuki Kenai, Tadashige Kano, Hisae Mori, Osamu Nagano, Seiko Hasegawa, Akira Inoue, Yasushi Nagatomo, Shinji Onoue, Manabu Sato, Soichiro Yasuda

**Affiliations:** 1 Neurosurgery, Nagoya Radiosurgery Center, Nagoya Kyoritsu Hospital, Nagoya, JPN; 2 Neurosurgery, Komaki City Hospital, Komaki, JPN; 3 Neurosurgery, Osaka City General Hospital, Osaka, JPN; 4 Neurosurgery, Yokohama Rosai Hospital, Yokohama, JPN; 5 Neurosurgery, Mito Gammahouse, Katsuta Hospital, Hitachinaka, JPN; 6 Neurosurgery, Tokyo Women's Medical University Hospital, Tokyo, JPN; 7 Neurosurgery, Nagatomi Neurosurgical Hospital, Oita, JPN; 8 Neurosurgery, Kano Neurosurgery Clinic, Maebashi, JPN; 9 Neurosurgery, National Cerebral and Cardiovascular Center, Suita, JPN; 10 Neurosurgery, Chiba Cerebral and Cardiovascular Center, Ichihara, JPN; 11 Neurosurgery, Kuroishi General Hospital, Kuroishi, JPN; 12 Neurosurgery, Yamagata Prefectural Central Hospital, Yamagata, JPN; 13 Neurosurgery, Kouseikai Takai Hospital, Tenri, JPN; 14 Neurosurgery, Ehime Prefectural Central Hospital, Matsuyama, JPN; 15 Neurosurgery, Rakusai Shimizu Hospital, Kyoto, JPN; 16 Neurosurgery, Shiroyama Hospital, Habikino, JPN

**Keywords:** craniopharyngioma, radiosurgery, gamma knife

## Abstract

Objective

The optimal treatment for a craniopharyngioma has been controversial. Complete resection is ideal, but it has been difficult to obtain total resection in many cases because of intimate proximity to critical structures such as the optic pathway, hypothalamus, and pituitary gland. A growing number of studies have demonstrated the utility of radiosurgery in controlling residual or recurrent craniopharyngioma. However, most of them are small series. The aim of this multi-institutional study was to clarify the efficacy and safety of Gamma Knife (Elekta, Stockholm, Sweden) surgery for patients with a craniopharyngioma.

Methods

This was a multi-institutional retrospective study by 16 medical centers of the Japan Leksell Gamma Knife Society. Data on patients with craniopharyngiomas treated with Gamma Knife Surgery (GKS) between 1991 and 2013 were obtained from individual institutional review board-approved databases at each center. A total of 242 patients with craniopharyngioma were included in this study. The mean age of the patients was 41 (range, 3 to 86) years. The median follow-up time was 61.4 months (range, 3 to 180 months). The mean radiosurgery target volume was 3.1 ml (range, 0.03-22.3 ml), and the mean marginal dose was 11.4 Gy (range, 8-20.4 Gy).

Results

Two-hundred twenty patients were alive at the time of the last follow-up visit. The three-, five-, and 10-year overall survival rates after GKS were 95.4%, 92.5%, and 82.0%, respectively. The three-, five-, and 10-year progression-free survival rates after GKS were 73.1%, 62.2%, and 42.6% respectively. The rate of radiation-induced complications was 6.2%.

Conclusion

GKS is effective for controlling the tumor growth of craniopharyngiomas with an acceptable complication rate.

## Introduction

Craniopharyngioma is a rare benign tumor thought to be derived from embryological remnants of the primitive craniopharyngeal duct or Rathke’s pouch. The incidence of craniopharyngioma is reported to be 0.13 in 100000, accounting for approximately 1%-4% of all central nervous system (CNS) neoplasms in adults and 5%-10% in children. The age of onset demonstrates a bimodal peaked distribution of 5-15 and 45-55 years old [1-3].

The optimal treatment for craniopharyngioma remains controversial. Surgery is usually considered the treatment of choice. It offers rapid symptom relief and pathological confirmation, and when total resection is achieved, long-term local control can be expected [4]. However, despite the advances in microsurgical and endoscopic techniques, it is difficult to achieve total resection in more than a few cases because of the intimate relationship with surrounding critical structures such as the hypothalamus, optic apparatus, and pituitary gland [5-7]. Several studies have reported the usefulness of Gamma Knife Surgery (GKS) for controlling craniopharyngiomas with a reduction in the complication rate; however, most have been small series [8-14]. 

We herein report a multi-institutional study (the largest series to date) on the efficacy of GKS for craniopharyngiomas.

## Materials and methods

Patient characteristics

This was a multi-institutional retrospective study conducted by 16 hospitals of the Japan Leksell Gamma Knife Society. The data of patients with craniopharyngiomas treated with GKS between 1991 and 2013 were obtained from individual institutional review board-approved databases at each center. A total of 242 patients with craniopharyngiomas were included in this study. The patient characteristics are shown in Table 1.

**Table 1:** Demographics of patients with craniopharyngioma GKS = Gamma Knife Surgery, KPS = Karnofsky Performance Status, VA = Visual Acuity, VF = Visual Defect

	Value	Range	%
Age at GKS (yrs)			
mean	41	3-86	
under 16	40		16.5
Gender			
male	127		52.5
female	115		47.5
Operation			
total resection	49		20.2
partial resection	182		75.2
biopsy	11		4.5
Previous treatment			
previous multiple operations	71		29.8
previous radiation	9		3.7
KPS			
80≦	219		90.5
Tumor nature			
solid	110		454.4
cystic	68		28.1
mixed	64		26.4
Tumor volume (ml)			
mean	3.1	0.03-22.3	
Tumor involvement site			
sellae-supra sellae	189		78.1
pro sellae	19		7.9
retro sellae	9		3.7
3rd ventricle	45		18.6
Visual disturbance			
VA decrease	132		54.5
VF defect	112		46.3
Pituitary function			
hypopituitarism	133		55
diabetes insipidus	100		41.3

All patients underwent surgical resection (including biopsy cases) before GKS and had histological confirmation of a craniopharyngioma. The mean duration between the diagnosis and the first GKS was 33.7 months (range 0-450). The first operation was total resection in 48 (19.8%), partial resection in 182 (75.2%), and biopsy in 11 (4.5%). Nine patients (3.7%) underwent conventional fractionated radiotherapy before the first GKS.

The nature of the tumor was solid in 110 (45.5%), cystic in 68 (28.1%), and mixed in 64 (26.4%) cases. One-hundred thirty-two patients (54.5%) had visual disturbance, and 153 (63.2%) patients had hypopituitarism at the first GKS. The mean age of the patient was 41.0 years (range 3-86 years old). Of the 242 patients, 40 (16.5%) were under 16 years old. The median Karnofsky Performance Status (KPS) score at the first GKS was 90.

The target tumor involvement site at the first GKS was sellae and/or supra sellae in 189 (78.1%), pro sellae in 19 (7.9%), retro sellae in 9 (3.7%), and third ventricle in 45 (18.6%).

Radiosurgery technique

Stereotactic radiosurgery was conducted using a Leksell Gamma Knife model B, C, 4C, and Perfexion (Elekta AB, Stockholm, Sweden) at each participating center.

A Leksell model G stereotactic coordinate frame (Elekta AB) was fixed to the patient’s head under local anesthesia supplemented by intramuscular and/or intravenous sedation. Stereotactic magnetic resonance imaging (MRI) was performed to define the tumor location and shape. Images were transferred via an ethernet connection to the Gamma Knife computer workstation (KULA system and GammaPlan; Elekta AB), where treatment planning was done. Contrast-enhanced MRI was used for the identification of the irregular borders of the neoplasm. The choice of the marginal irradiation dose depended on the tumor volume and the spatial relationship between the tumor and the adjacent anatomical structures, especially the visual pathway. After dose planning, patients were treated with Gamma Knife Units B, C, 4C, or Perfexion (AB Elekta) at participating centers.

The median tumor volume was 3.1 cm^3^(range, 0.03-22.3 cm^3^). The median maximum dose was 21.8 Gy (range, 8-36 Gy), the median margin dose was 11.4 Gy (range, 8-20.4 Gy), and the amount irradiated to the optic pathway varied from 1.6 to 14 Gy (median, 7.3 Gy).

Follow-up

All patients underwent both clinical and radiological evaluations at each participating institute, including a neurological examination, visual examination, hormonal evaluation, and MRI regularly performed at three- to six-month intervals after GKS. At each imaging analysis, the tumor response was categorized into one of four results: complete response (CR, tumor disappearance), partial response (PR, >50% reduction of the tumor volume), no change (SD, stable disease), and progression (PG, >25% increase in tumor volume). If new tumors or progression of the treated tumors were noted during follow-up, appropriate additional treatment, including repeat GKS, other types of radiation, or surgical resection, were offered.

Statistical analyses

The overall survival (OS) and progression-free survival (PFS) after the first GKS was calculated using the Kaplan-Meier estimator. The log-rank test was utilized to compare the survival differences between groups. To analyze factors that correlated with the PFS, we assessed the following data: age, sex (male vs. female), tumor nature, tumor location, tumor volume, maximum dose, and marginal dose. P-values <0.05 were considered statistically significant. The statistical analysis was performed using the JMP software program, version 7 (SAS Institute Inc., Cary, North Carolina).

## Results

The median clinical follow-up period was 61.4 months (range, 3-180 months), and the median radiological follow-up period was 57.3 months (range, 3-205 months).

The OS

**Figure 1:**
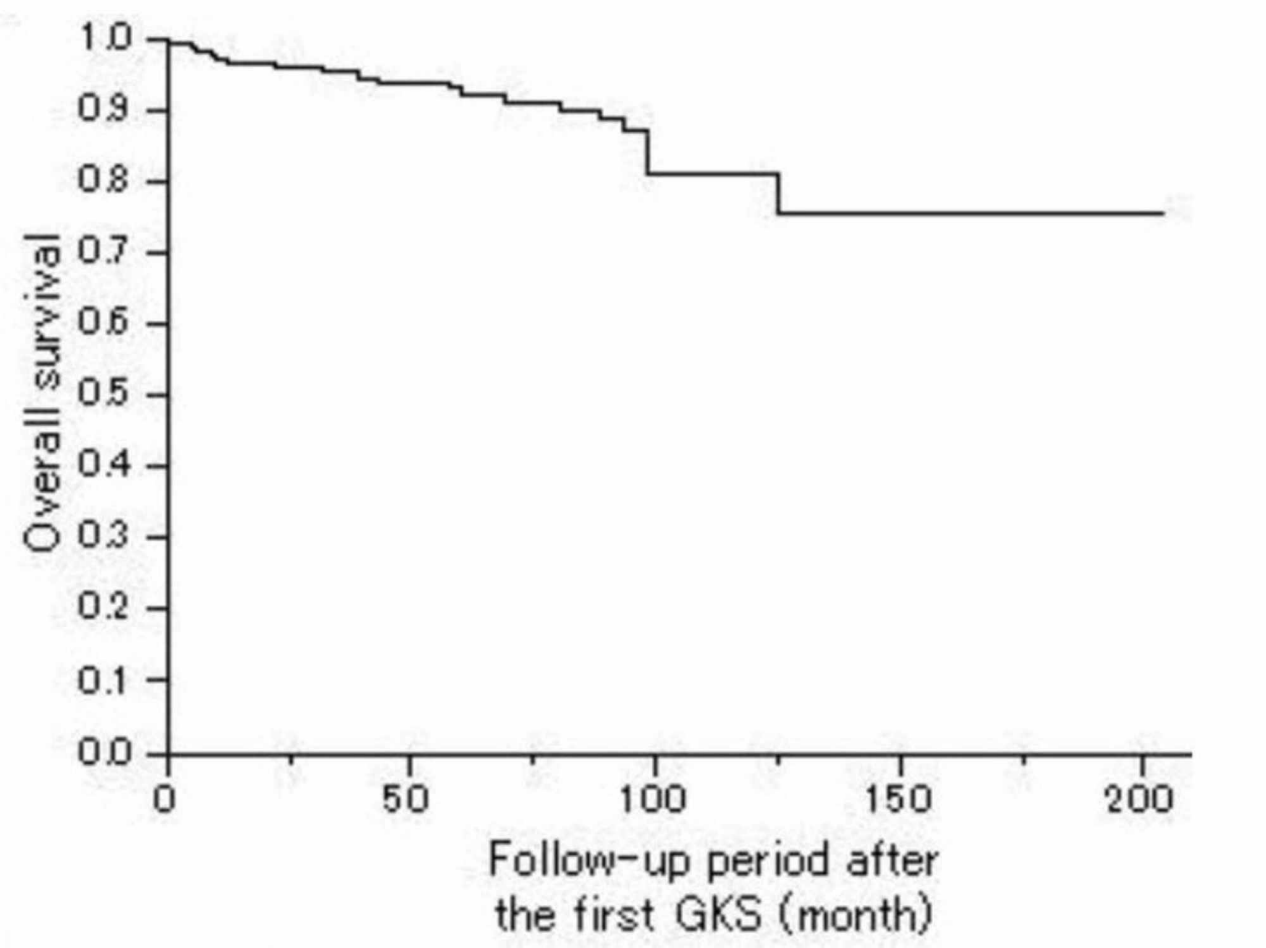
Overall survival Kaplan-Meier curve showing the OS of 242 patients with a craniopharyngioma. The actual three-, five-, and 10-year OS rates after the first GKS was 95.4%, 62.5%, and 82.0%, respectively. OS = Overall Survival, GKS = Gamma Knife Surgery

Two-hundred twenty patients (90.9%) were alive at the last follow-up time visit, with 22 (9.1%) deaths. Of these 22 patients, 11 (4.5%) died of tumor progression. The remaining 11 died of another reason such as lung cancer, pneumonia, and renal failure. The three-, five-, and 10-year overall survival rates after the first GKS were 95.4%, 92.5%, and 82.0%, respectively (Figure 1).

**Figure 2:**
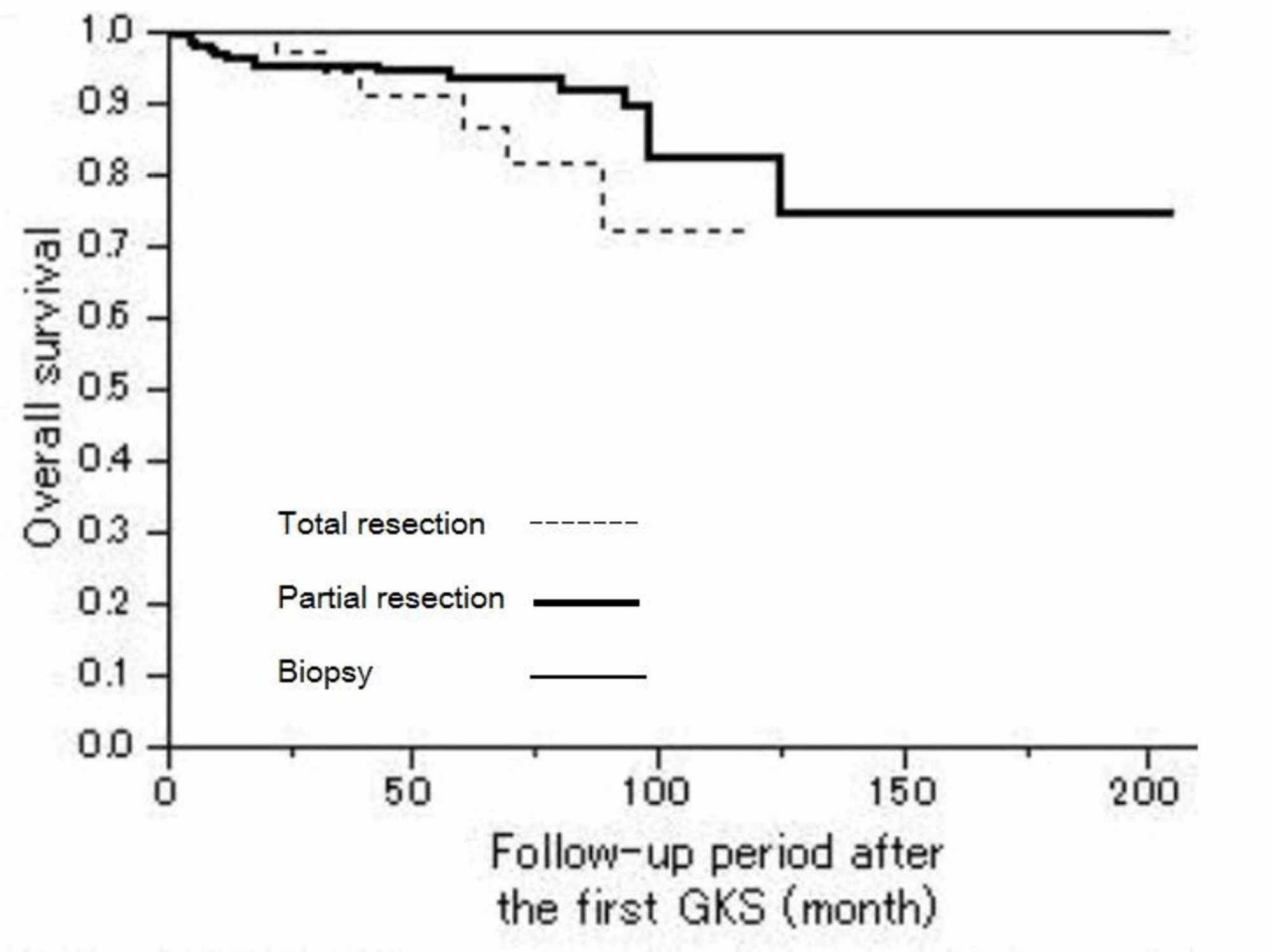
Overall survival depending on the type of the first operation Kaplan-Meier OS curves showed no statistically significant difference in relation to the types of the first operation (Log-rank, P = 0.1629). P < 0.05 statistically significant, OS = Overall Survival, GKS = Gamma Knife Surgery

The overall survival showed no statistically significant difference according to the types of the first surgical operation (Figure 2).

The median KPS score at the last follow-up visit was 90.

Tumor control

**Figure 3:**
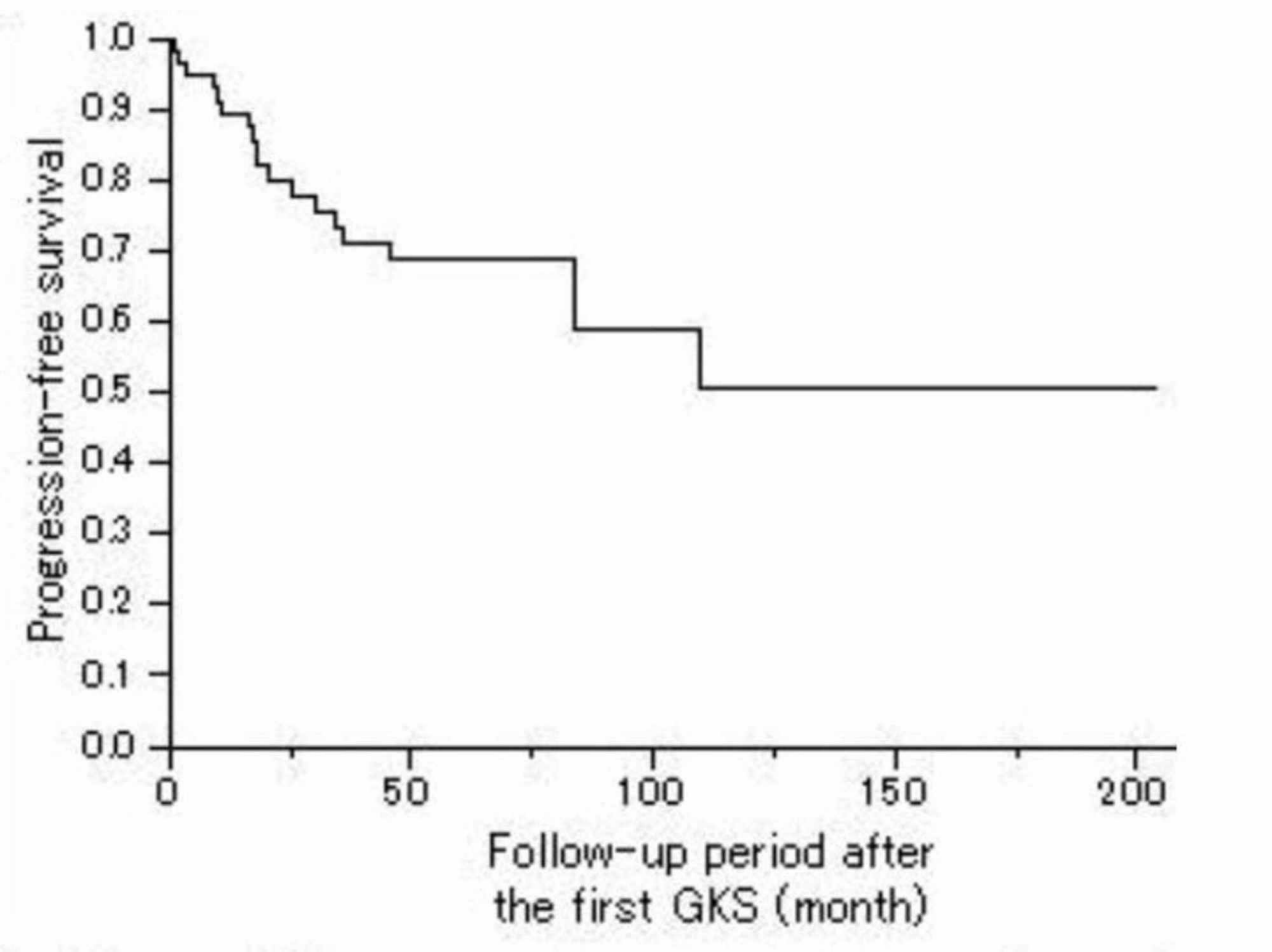
Progression-free survival Kaplan-Meier curve showing the PFS of 242 patients with a craniopharyngioma. The actual three-, five-, and 10-year PFS rates after the first GKS was 73.1%, 62.2%, and 42.6%, respectively. PFS = Progression-Free Survival, GKS = Gamma Knife Surgery

The three-, five-, and 10-year PFS rates after the first GKS were 73.1%, 62.2%, and 42.6%, respectively (Figure 3).

**Figure 4:**
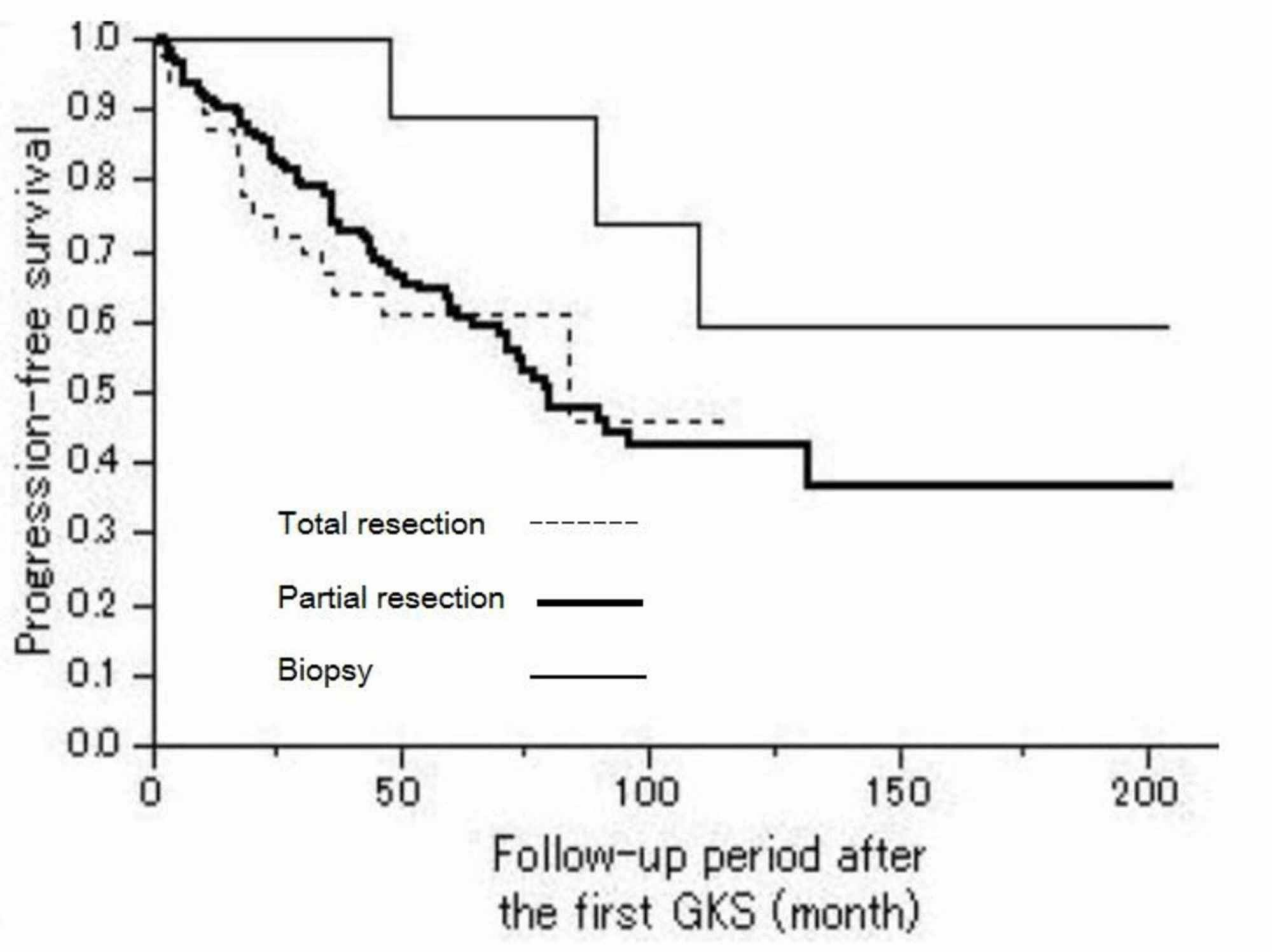
Progression-free survival depending on the types of the first operation Kaplan-Meier PFS curves showed no statistically significant difference in relation to the types of the first operation (Log-rank, P = 0.2626). P < 0.05 statistically significant, PFS = Progression-Free Survival, GKS = Gamma Knife Surgery

 The PFS showed no statistically significant difference according to the types of the first surgical operation (Figure 4).

A univariate analysis showed that the tumor volume and tumor location (extending to the third ventricle) were significantly correlated with the PFS (Table 2).

**Table 2:** Univariate analysis of prognostic factors related to PFS P < 0.05 statistically significant; PFS = Progression-Free Survival

Factor	Univariate P-value
Sex	0.8201
Age ( < 16)	0.7652
Resection (total resection or not)	0.7568
Tumor volume	0.0102*
Tumor nature	0.2721
Location (extension to third ventricle)	0.0317*
Marginal dose	0.5134
Maximum dose	0.7674

**Figure 5:**
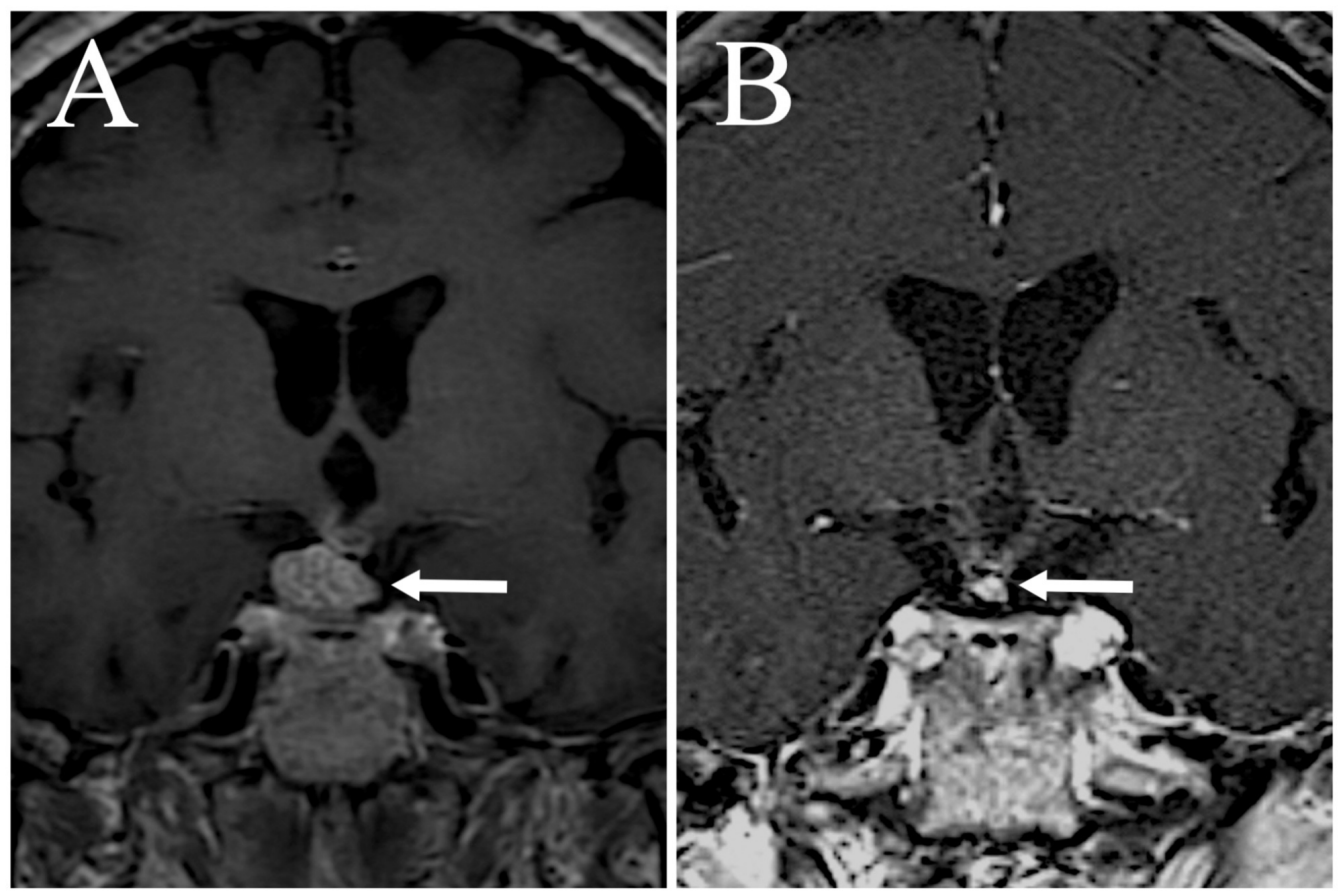
Illustrative case A 66-year-old female. Coronal T1-weighted magnetic resonance imaging (MRI) with gadolinium (Gd) enhancement depicting a recurrent craniopharyngioma during GKS (A). The recurrent tumor was treated with a margin dose of 11 Gy. Follow-up MRI with Gd enhancement performed 40 months after GKS showing tumor regression (B). GKS = Gamma Knife Surgery; MRI = Magnetic Resonance Imaging

The MRI findings of the response of the tumor treated by the first GKS were CR in 19 (7.9%), PR in 82 (33.9%), NC in 47 (19.4%), and PG in 74 (30.6%). In addition to local recurrence, new tumors developed outside of the irradiated area in 22 (9.1%) cases. Figure 5 depicts an illustrative case.

Patient management

In the course of tumor management, nearly half of the cases required multiple treatments. The total number of Gamma Knife procedures applied was one in 139 cases (16.1%), two in 59 cases (24.4%), three in 28 cases (11.6%), and ≥ four in 11 cases (4.5%). The total number of surgeries performed was one in 140 cases (16.5%), two in 60 cases (24.8%), three in 28 cases (11.6%), and ≥ four in 11 cases (11.6%). The treatment for a recurrent tumor after GKS was a repeat GKS in 43 (17.8%), an operation in 51 (21.1%), and irradiation without GKS in 14 (5.8%).

The hormonal function

One-hundred fifty-three patients (63.2%) had anterior pituitary dysfunction, and 100 (41.3%) had diabetes insipidus (DI) at the time of the first GKS. The anterior pituitary function at the last follow-up visit improved in one and deteriorated in 10 as compared with the function at the first GKS. Fifteen patients showed deterioration of the posterior pituitary function, whereas two who had DI were able to discontinue the medication at the last follow-up visit. Of the 15 patients who presented new DI, 12 cases developed after additional surgical resection.

The visual function

One-hundred thirty-two patients (62.8%) had disturbance of visual acuity, and 112 (46.2%) had visual field defects at the time of the first GKS. At the last follow-up visit, the visual acuity was improved in seven cases, and visual field defects were improved in 18 cases, whereas eight patients showed deterioration of the visual acuity and eight patients showed deterioration of their visual field.

Adverse effects

Fifteen patients developed radiation-induced side effects, including visual deterioration in four, deterioration of the anterior pituitary function in eight, DI in three, hypothalamic dysfunction (obesity) in one, brain edema in one, and hydrocephalus in one. The rate of radiation-induced complications was 6.2%.

## Discussion

While a craniopharyngioma is histologically benign, corresponding to World Health Organization (WHO) grade I, as it grows, it begins to aggressively interact with the surrounding critical organs, such as the optic pathway, pituitary gland, vascular structures, and hypothalamus, causing impairment. The optimal treatment for a craniopharyngioma has been controversial. The difficulty in planning the treatment strategy for craniopharyngioma is mostly due to the intimate anatomical relationship with surrounding critical structures [5-7]. 

Because the life expectancy of the patients harboring this tumor may be extended with appropriate management, complications associated with treatment should be reduced as much as possible. Aggressive surgery offers a greater chance of achieving total resection, which leads to a favorable local control rate, but it may cause unacceptable complications such as visual disturbance, pituitary dysfunction, and cognitive disturbance [15-17]. The treatment strategy should be determined after considering that the tumor may recur after the operation even if gross total resection is achieved [18-19].

Fractionated external-beam radiation following partial removal is effective for controlling tumor growth and improving patient survival [20-21]. However, there are still issues remaining to be addressed, such as tumor regrowth and late-onset radiation-related visual, hypothalamic, and endocrine complications [22-23].

Recently, with the development of stereotactic radiation techniques, partial resection followed by stereotactic irradiation has been accepted as a viable treatment approach for craniopharyngioma (Table 3) [8-14,24-27].

**Table 3:** Representative reported results on stereotactic radiosurgery and stereotactic radiotherapy GKS = Gamma Knife Surgery, CKS = CyberKnife Surgery, FSRT = Fractionated Stereotactic Radiation Therapy, F/U = Follow-Up, N/A = Not Applicable

Author & year	Radiation therapy	No. of patients	Mean tumor volume (ml)	Mean marginal dose (Gy)	F/U (month)	Tumor control rate (%)	Morbidity (%)
Ulfarsson et al. 2002	GKS	21	8	5	13	36	19
Yomo et al. 2009	GKS	18	3.5	11.5	26	94	0
Niranjan et al. 2010	GKS	46	1	13	62	68	17.4
Lee et al. 2014	GKS	137	5.5	12	53	69	11.7
Dho et al. 2018	GKS	35	1.4	15 (single-session), 18( fractionated)	71.9	63 (5 yrs)	N/A
Iwata et al. 2012	CKS	43	0.5 (single-session), 2.2(fractionated)	14 (single-session), 16-25 (fractionated)	40	85 (5 yrs)	2.3
Harrabi et al. 2014	FSRT	55	31.2(PTV)	52.2 (1.8-2 per day	128	92 (10 yrs)	3.6
Present study	GKS	242	3.1	11.4	61	69	6.2

This treatment strategy is supported by the fact that this tumor responds relatively well to irradiation [20-21]. The drawback of radiation is the possibility of its toxic effect on surrounding normal structures, but this adverse effect can be reduced by precise stereotactic irradiation of the target. Several studies have described the role of GKS for controlling craniopharyngiomas [8-14]. Hasegawa et al. reported that the five- and 10-year PFS rates were 62% and 52%, respectively, and Lee et al. described their 20-year experience with GKS for craniopharyngioma and reported the volume of the treated tumor as a significant prognostic factor for the PFS [8,13]. Dho et al. demonstrated that the location of the tumor, the distance between the optic nerve and tumor, and the radiation dose were statistically significant, with an overall response rate [10]. Kobayashi et al. reported that a prescribed dose exceeding 13.2 Gy could be expected to achieve a better PFS than lower doses [14]. As in these studies, several other reports also suggest the efficacy of GKS for the management of this tumor. However, most of those studies were small series because of the low incidence of this disease. We, therefore, performed a multi-institutional study in our evaluation of 242 cases, making this the largest series to date.

In the present study, we demonstrated the effectiveness of GKS for controlling residual and/or recurrent craniopharyngiomas, and, at the same time, showed the points to consider for achieving a better clinical course by this treatment strategy, namely, performing partial resection followed by GKS. The three-, five-, and 10-year OS rates after GKS were 95.4%, 92.6%, and 82.1%, respectively, whereas the PFS rates were 71.7%, 60.3%, and 41.9%, respectively. The OS rate seems to be acceptable, but the PFS rate leaves room for improvement.

While a higher radiation dose is expected to yield a stronger effect on the tumors, it may also carry an increased risk of adverse effects on the surrounding normal tissue. One advantage of GKS is the steep drop-off in radiation, which delivers sufficient irradiation to the target tissue while sparing the surrounding normal tissue. However, this advantage can only occur when the whole area of the tumor is adequately and precisely irradiated. Because if there is a part of a tumor that is not properly irradiated, it may receive an insufficient amount of irradiation to successfully control disease progression. Given that a sizeable portion of recurrence after GKS occurred in the outfield area of irradiation in the present study, it is important to properly recognize the area where the tumor exists, with consideration of the steep irradiation drop-off, in order to improve the PFS rate with GKS. Insufficient targeting might be one reason why the prescribed dose was not a significant factor influencing the PFS in this study.

Univariate analysis revealed the tumor volume and third ventricle involvement of the target tumor to be significant factors that influence the PFS. When the tumor grows, extending into the third ventricle, a larger area of the hypothalamus tends to become involved with the tumor. To prevent serious impairment of the hypothalamus, surgical manipulation at this site tends to be less aggressive, leading to a greater amount of residual tumor in this area, some of which might be difficult to identify during targeting due to surgical artifacts and other reasons on MRI. Furthermore, the locational proximity to the optic tract and the important function of the hypothalamus requires limiting the irradiation dose at the target tumor at this site. Generally, when tumors are located widely with an irregular shape, they tend to require more effort to identify the target’s margin precisely. The difficulty and insufficient identification of the target tumor margin might contribute to an insufficient PFS rate. It goes without saying that, when developing a treatment plan for stereotactic irradiation, it is important to evaluate the extent of the target tumor location meticulously with imaging studies, such as MRI, taken not only at the time of planning but also before and after surgery on follow-up imaging and discuss the extent of the tumor border with the surgeons.

If the tumor is attached to an organ at risk (OAR), such as the optic pathway, which is vulnerable to radiation, it is difficult to deliver the ideal radiation dose to the target and avoid adverse effects, like radiation-induced optic neuropathy. Hasegawa et al. reported that radiation to the optic nerve exceeding 14 Gy might cause visual disturbance, even when delivered to a short segment of the nerve, and 10 Gy to a long segment may lead to optic neuropathy [13]. This report indicates that the optic pathway is more tolerable than previously thought, but when a tumor is close to or attaches to the optic pathway, the irradiation dose must be reduced to a dose that is too low to control the tumor. In addition, if the tumor is large, the irradiation dose needs to be kept low in order to avoid toxic effects on the surrounding normal tissue. In the present study, several cases required treatment of the target tumor with a reduced dose because of these reasons. This also seemed to contribute to an insufficient PFS rate.

To deliver sufficient irradiation to the target tumor while minimizing the toxic effects on the surrounding normal tissue is the key to better controlling a tumor. The adverse effects of irradiation on normal tissue can be reduced depending on the fraction. With the advent of new-generation GKS-the Gamma Knife Icon, which adopts mask fixation, fractionated GKS has become much more widely available. Fractionation is expected to reduce adverse effects on the optic pathway, hormonal function, and hypothalamic function. In the near future, fractionated irradiation might become the standard for GKS in treating craniopharyngioma.

In this study, the rate of radiation-induced complications was 6.2%. This number is similar to that in previously reported series and seems to be relatively small, considering the critical location of the tumor. This is probably due to the accurate and relatively low-dose delivery of radiation to treat the tumors. However, at the final follow-up visit, more patients than this rate presented with a deterioration of the visual and hormonal function. While tumor progression is the primary factor that causes the impairment of important functions, such as visual, hormonal, and cognitive functions, the adverse effects of the treatments themselves also negatively affect these functions. In many cases in the present study, multiple treatments, including repeat surgery and repeat GKS, were employed to control the tumors. The longer the treatment course, the more patients tend to suffer from poor dysfunction caused by not only tumor progression but also the adverse effect of the treatments. Once important functions are impaired, it is almost impossible to restore them. Therefore, treatment should be performed as total management of the tumor, taking advantage of various modalities.

In pediatric patients harboring this tumor, the generation of secondary disorders, such as malignant tumor and vascular degeneration, is sometimes problematic in the long-term follow-up [22-23,28-29]. In addition, some studies have reported malignant changes in craniopharyngiomas during the course of treatment, which were suspected to have been caused by irradiation [30].

Because this tumor is pathologically benign, adverse effects in the long term should be discreetly considered, especially in pediatric patients. Avoiding irradiating, an excessively broad area is expected to reduce the early and late toxic effects on the surrounding organs and the possible generation of pathological conditions. Precise irradiation of the tumor by GKS following surgical resection with minimal invasion, which preserves the neurological and hormonal function, is key to achieving a better overall clinical course in patients with a craniopharyngioma.

Study limitations

Because this was a retrospective multi-institutional study, the criteria of SRS or radiosurgical techniques may have differed among institutes.

## Conclusions

We herein described a multi-institutional study on the efficacy of GKS for craniopharyngiomas in our evaluation of 242 cases, the largest such series to date. In this study, we reported the following: 1) The three-, five-, and 10-year overall survival rates after GKS were 95.4%, 92.5%, and 82.0%, respectively. 2) The three-, five-, and 10-year PFS rates after GKS were 73.1%, 62.2%, and 42.6%, respectively. 3) Univariate analysis showed that the tumor volume and tumor location (extending to the third ventricle) were significantly correlated with the PFS. 4) The rate of radiation-induced complications was 6.2%. GKS is effective in controlling the tumor growth of residual or recurrent craniopharyngiomas with an acceptable complication rate.
